# Do current biomass equations for *Alnus glutinosa* and* Betula pubescens* misestimate carbon stocks at peatland sites?

**DOI:** 10.1186/s13021-025-00360-x

**Published:** 2026-01-03

**Authors:** Henriette Gercken, Marius Möller, Ana Lucia Mendez Cartin, Judith Bielefeldt, Emilia Wolfram, Nicole Wellbrock, Julian Gärtner, Cornelius Oertel

**Affiliations:** 1 Institute of Forest Ecosystems, Thuenen Institute, Alfred-Möller-Str. 1, Haus 41/42, Eberswalde, 16225 Brandenburg Germany; 2 Forest Science and Technology Centre of Catalonia (CTFC), St. Llorenc de Morunys, km 2 (direc. Port del Comte), Solsona, 25280 Catalonia Spain; 3 Eberswalde University of Sustainable Development (HNEE), Schicklerstraße 5, Eberswalde, 16225 Brandenburg Germany

**Keywords:** Biomass, Peatland forests, Carbon stock, Alnus glutinosa, Betula pubescens, Growth dynamics

## Abstract

Accurate estimation of forest carbon stocks is essential for climate change mitigation, particularly in peatland ecosystems known for their high soil organic carbon content. However, biomass equations currently used in Germany, such as the ^“^regular” biomass equation of the National Forest Inventory integrated in the *TapeS* R package, are primarily calibrated for mineral soil sites and may misestimate biomass in peatland forests. This study evaluates the applicability of existing biomass equations for* Alnus glutinosa* and *Betula pubescens* in forested peatlands across Germany by comparing estimates of the biomass equation of the National Forest Inventory with a set of alternative allometric functions, including peatland-specific equations. Using data from 65 forests at peatland and 1266 forests at mineral soil sites, we assessed tree growth patterns, aboveground biomass, and carbon stocks. Results indicate significant differences in growth dynamics between peatland and mineral sites, with trees at peatland sites exhibiting lower heights and biomass at a given diameter. Despite this, stand level carbon estimates by the standard biomass equation of the National Forest Inventory aligned closely with the mean of all equations for both species and did not show a consistent bias, although it overestimated individual tree biomass for *Betula pubescens*. Notably, peatland-specific functions show high variability and no clear advantage over the biomass equation of the National Forest Inventory. We conclude that while the equation of the National Forest Inventory currently provides robust estimates for the carbon stock of peatland forests in Germany on stand level for *Betula pubescens* and *Alnus glutinosa*, future recalibration may be needed as restoration efforts and climate change alter site conditions. For local-scale applications, especially in intact or rewetted peatlands, site-adapted equations are recommended to account for the high spatial heterogeneity and complex growth dynamics of these ecosystems.

**Supplementary Information:**

The online version contains supplementary material available at 10.1186/s13021-025-00360-x.

## Introduction

Climate change poses an increasing threat to the global ecosystem and human well-being, by directly impacting forest growth and provision of ecosystem services [54, 58, 64]. Therefore, effective mitigation and adaptation measures are urgently needed to prevent its worst impacts [54, 58, 87]. Multiple international conventions have urged signing countries to minimize the causes of climate change by tackling pollution sources and enhancing forest capacity to sequestrate carbon (*C*) [87, 88]. In the European Union, member states are obliged to make considerable efforts to reduce greenhouse gas (*GHG*) emissions, as they aim to achieve climate neutrality by 2050 [13, 87, 88]. Forests are essential for *GHG* reduction because of their crucial role in global carbon dynamics, as they not only absorb billions of tons of CO_2_ annually but also constitute one of the worlds largest terrestrial carbon pools, storing between 70–90% of terrestrial biomass [28, 51]. Therefore, forest policies in the European Union aim for both, the increment of forest cover and carbon sequestration through forest management [18].

In addition, there is a growing societal demand for forests to provide ecosystem services beyond timber production[15, 20, 59], This has led to a revaluation of forests regulation services, such as carbon sequestration [15, 20, 59]. However, the ability of a forest to provide ecosystem services strongly depends on the applied management regime [15, 60, 79]. In this regard, close-to-nature management practices and ecosystem conservation have proven particularly effective in increasing the *C* sink function of forests while simultaneously improving the provision of other ecosystem services demanded by society [15, 51, 59, 60]. This has led to a general shift from one-purpose, wood production-oriented forestry towards multi-purpose, integrated forest management [20], which can favor undervalued forests, such as peatlands and *Betulas spp.* and *Alnus spp.* dominated forests.

Forested peatlands can store twice as much carbon as regular forests by accumulating peat from dead plant material that removed CO_2_ from the atmosphere [10, 30, 46, 82, 89]. Thus, the enormous carbon sequestration potential of peatlands results from their thick peat layer that comprises more than 30% of soil organic matter (*SOM*), containing approximately 50% of soil organic carbon (*SOC*) [2, 17, 47]. Although the role of peat *SOC* in mitigating climate change is becoming increasingly important, the above-ground carbon dynamics of peatland forest communities have been widely disregarded in the past [5, 57, 62, 82]. Therefore, to track and manage how forests affect climate change, accurate ways of quantifying tree biomass are needed.

Allometric functions are a well-established tool in forest yield science to describe growth dynamics and quantify tree stocks. Since they are used primarily to plan timber production, they typically focus on the main commercially relevant species and estimate volume rather than biomass [95]. In light of climate change as a growing threat and stricter international obligations for *GHG* reporting, functions estimating single-tree biomass have become more relevant [7, 39, 87, 88]. Yet, for forested peatlands most studies and models focus primarily on carbon stored in the peat-body itself [5, 57], where the wet, acidic and extremely nutrient-poor soil conditions lead to very low productivity, rendering these stands nearly irrelevant for timber production and inaccessibly for timber harvest [38, 53, 61, 82]. Additionally, available research on forested peatlands comes primarily from countries in the boreal region, where a large proportion of peatlands used for forestry are drained for this purpose [82].

Therefore, biomass functions for typical forest communities of intact peatlands are hardly available and, if so, they fail to represent peatland growing conditions.

In temperate regions, currently ongoing peatland re-naturation efforts are likely to cause the re-establishment of site-adapted peat forest communities [7, 16] which are typically dominated by water-tolerant species such as *Alnus glutinosa* and *Betula pubescens* [16]. Most biomass equations for these species are found for Scandinavian countries such as Finland [32–34, 65], Sweden [43, 45] and Norway [8, 81]. In temperate regions, peatland-specific equations were found for Poland [94], the USA [42] and England [29]. Yet, there is an absence of available functions that explicitly represent the growth of peatland forest communities in Germany. Therefore, biomass in peatland forests is estimated using the “regular” biomass equation of the German National Forest Inventory (*NFI*) which is also used to account for the carbon balance of the country’s forest sector (hereafter referred to as equation 41, *DEU*) [24, 68, 90]. This overall aboveground biomass equation, in the case of *Betula pubescens* (*B. pubescens*) and *Alnus glutinosa* (*A. glutinosa*), uses coefficients for a whole group of species, instead of calculating species-specific coefficients for each of them [67, 90, 91]. These coefficients are parametrised based on pseudo-observations originating from yield tables by Grundner and Schwappach [26], which are not only outdated but were also developed for mineral sites [26, 63, 67]. Therefore, applying this biomass function to peatland forests can carry uncertainty on the accuracy of its predictions. However, its comparability with past and future National Forest Inventories (*NFI*), National Forest Soil Inventories (*NFSI*), and National Greenhouse Gas Reporting argues in favour of its use. Still, other biomass functions have been found to estimate biomass of *B. pubescens* in Germany, but they focus on mineral soils of a specific region [3]. Similarly to A. glutinosa, whose main base of species-specific biomass estimations are region specific yield tables developed by Lockow et al.[56] for Northeast Germany. Therefore, the resulting lack of extrapolation capacity may not allow for these equations or tables to be used on a broader scale.

The lack of a species-specific equation parametrised on a national level limits the current peatland forest carbon estimation in Germany, as, to our knowledge, none of the available functions are developed or validated with real peatland forest biomass data, nor tested with other peatland biomass specific equations to determine their predictive capacity. The increasing importance of peatland forests *C* stocks and the simultaneous uncertainty associated with current *C* assessments in these ecosystems create a need to increase the accuracy of biomass estimations [7]. Therefore, our study aims to assess the predictive capacity of the 41, *DEU* function currently used to calculate *C* stocks in Germany, by comparing its predictions with those of peatland- and species-specific equations parametrised for several extratropical areas. We aim to answer the research question of whether the 41, *DEU* equation is capable of predicting biomass stocks similar to peatland-specific equations. We hypothesised that (1) the growth dynamics of forest communities differ significantly between peatland and mineral sites and (2) hence the existing 41, *DEU* cannot be applied to peatland forests of *A. glutinosa* and *B. pubescens* without adjustments. Further, we assume that (3) the 41, *DEU* equation’s predictors are dissimilar to the peatland-specific equations, causing the overestimation of carbon stocks compared to equations created for peatlands in temperate regions and the overall mean of all peatland- and species-specific equations.

## Methods

### Study area

The study includes sites throughout the entire territory of Germany, a country situated in Central Europe between latitudes 47° and 55° N and longitudes 5° and 15° E. Germany features a temperate seasonal climate, with generally humid conditions, mild to warm summers, and cool winters. Annual precipitation varies regionally, but typically ranges between 500 and 1000 mm, supporting diverse forest types, ranging from the *Picea abies* or *Abies alba* dominated mountain forests in subalpine regions over deciduous forests dominated by *Fagus sylvatica* in central Germany to *Alnus glutinosa* and *Alnus incana*, *Betula pubescens* or *Pinus sylvestris* peatland forests concentrated in the north German plain [77, 86]. Forests cover around 30% of the country’s surface area and consists mainly of of temperate broad-leafed and mixed forests and managed woodlands [69, 92]. However, due to commercial purposes, the most common species are currently *Pinus sylvestris* and *Picea abies*, followed by *Fagus sylvatica* and *Quercus spp.* [69]. The main coniferous species are also found in peatlands, where they represent the predominant tree species together with *Betula pubescens* and *Alnus glutinosa* [16]. Peatlands in Germany cover approximately 1.8 million hectares, which represents approximately 5% of the country’s surface area [7], where about 15% are forested [7].

This study focuses specifically on plots throughout Germany that contain *A. glutinosa* and *B. pubescens*, which occur at peatland as well as mineral soil sites. *A. glutinosa* account for 2.6% of Germany’s forest cover [84] and thrive at sites with high water saturation up to periodic flooding and water stagnation, because of their high tolerance to waterlogging [21, 38, 78]. Due of their moderate requirements in terms of base and nutrient supply, they are displaced by *B. pubescens* in heavily acidified, nutrient-poor locations [21, 78]. Trees of the botanical genus *Betula* cover 4.7% of the countries forest area [84], whereby the distribution of *B. pubescens* concentrates on acidic peatland sites [6, 78].

### Selection of biomass equations

In order to compare the predictive capacity of 41, *DEU* to the results of other species- and peatland specific biomass equations we carried out a literature review to collect allometric functions for *A. glutinosa* and *B. pubsecens*. Since water table fluctuations are the main driver of tree growth dynamics at peatland forests [4, 53, 66, 71, 72] and drainage promotes tree growth [53, 66], we assume that tree growth will become increasingly detached from the hydrological regime and approximate the growth dynamics at mineral sites. Therefore, we have included equations that originate from peatlands and mineral sites to take into account the fact that most peatlands in Germany have been drained [7].

The following keywords were used for the search: “biomass function alnus glutinosa”, “biomass equation alnus glutinosa”, “biomass alnus glutinosa”, “biomass allocation alnus glutinosa”, “biomass function betula pubescens”, “biomass equation betula pubescens”, “biomass betula pubescens” “biomass allocation betula pubescens”. Publications were included in the analysis only if (1) the relevant keywords appeared in the title, abstract, or listed keywords, (2) the equations originate from extratropical regions, and (3) they presented equations and parameters to estimate the woody above-ground biomass of individual trees (*WAG*) based on diameter at breast height (*DBH*, *cm*) and height (*H*, *m*). The tree compartment “foliage” had to be excluded from the aboveground biomass equations sourced from the literature review to ensure better comparability with equation 41, *DEU*. Therefore, we excluded those functions that included foliage in their calculation, as 41, *DEU* only accounts for the aboveground woody tree compartments. Additionally, we only considered equations that were species specific for *A. glutinosa* and *B. pubsecens*.

This selection returned a final set of seven publications for *A. glutinosa* [29, 43, 49, 55, 74, 76, 94] and 11 publications concerning *B. pubescens* [3, 8, 11, 27, 31–34, 44, 65, 81], that provided a biomass equation to calculate the total *WAG* or equations for the trees woody compartments that were then summarised to extrapolate the *WAG* (Table 1).

**Table 1:** Overview of biomass equations sourced from literature review with their reference (“reference”) for *Betula pubescens* and *Alnus glutinosa* (“species”).

ID	Species	Country	Variables	Compartment	Peat	Reference
1, 1, *DEU*	Betula	Germany	*DBH*	*WAG*	min	Albert et al. [3]
1, 2, *DEU*	Betula	Germany	*DBH*,*H*	*WAG*	min	Albert et al. [3]
6, *NOR*	Betula	Norway	*DBH*	*SW*^1^	min	Bollandsås et al. [8]
7, *EST*	Betula	Estonia	*DBH*	*SW*+*SWB*	min	Buht et al. [11]
8, *ESP*	Betula	Spain	*DBH*,*H*	*SWB + SWB + FWB*	min	Gómez-García et al. [27]
10, *ISL*	Betula	Island	*DBH*	* SWB + FWB*	min	Hunziker [31]
11, 2, *FIN*	Betula	Finland	*DBH*	*WAG*	peat	Hytönen and Saarsalmi [34]
19, *FIN*	Betula	Finland	*DBH*	* SW + FWB*	peat	Hytönen and Aro [32]
20, *FIN*	Betula	Finland	*DBH*	*WAG*	peat	Hytönen and Kaunisto [33]
15, *SWE*	Betula	Sweden	*DBH*	*SW + SWB + FWB *	min	Johansson [43]
26, *FIN*	Betula	Finland	*DBH*,*H*	*SW + SWB + FWB + STW*	mix	Repola [65]
29, 11, *NOR*	Betula	Norway	*DBH*	* SW + SWB + FWB*	min	Smith et al. [81]
29, 12, *NOR*	Betula	Norway	*DBH*,*H*	* SW + SWB + FWB*	min	Smith et al. [81]
14, *USA*	Alnus	USA	*DBH*	*SW + SWB*	min	Jenkins et al. [42]
23, 4, *LVA*	Alnus	Latvia	*DBH*,*H*	*WAG*	min	Liepiņš et al. [55]
23, 5, *LVA*	Alnus	Latvia	*DBH*,*H*	*WAG*	min	Liepiņš et al. [55]
24, 1, *GBR*	Alnus	United Kingdom	*DBH*	*WAG*	min	Hughes [29]
27, *ESP*	Alnus	Spain	*DBH*, *H*	*SW + FWB*	min	Ruiz-Peinado Gertrudix et al. [75]
28, *TUR*	Alnus	Turkey	*DBH*,*H*	*SW + SWB + FWB*	min	Saracoglu [76]
32, *SWE*	Alnus	Sweden	*DBH*	* SW + FWB*	mix	Johansson [44]
39, *POL*	Alnus	Poland	*DBH*	*SW + SWB + FWB*	min	Wojciech [94]
40, *POL*	Alnus	Poland	*DBH*	* SW + SWB + FWB*	peat	Wojciech [94]

$^1FWB$available but not included since it was provided as “crown” consisting of woody compartments and leaf massColumn “ID" refers to the subsequent number of the publication, number of equation (if there were multiple equations per publication) and the country (“country") the equation originates from. Variables required to apply the equation are presented in “variables". “Peat" shows the site type the equation was parametrised for, “min" being mineral sites, “peat" are peatland sites, and “mix" reffers to euqations that were developed with data from both site types. Information about the tree compartments used to calculate the are reported in “compartment" where “WAG" refers to the total woody aboveground biomass, “SW" is stemwood, “SWB" is stemwood bark, “STW" is stumpwood, “STB" is stumpwood bark and “FWB" is finewood. All equations as well as more detailed information for each function provided in .

### Data acquisition and processing

Data was obtained from the second *NFSI* and the Peatland Monitoring Program for Climate Protection - Forest project ($MoMoK-Wald$). The *NFSI* *II* soil data was sampled between 2006 and 2008 [93], while the corresponding stand data was collected between 2011 and 2012 [36]. The forest inventory at $MoMoK-Wald$ plots used in this study was carried out between 2021 and 2024 [23].

We extracted the plots located in peatlands from the totality of 1284 *NFSI* *II* and 47 $MoMoK-Wald$ plots. We selected only those plots that were defined as organic soils by the German soil classification system (*KA*5) [1] and that also aligned with the IPCC definition for peatland [17, 40]. The IPCC defines peat soils as soils that have an organic horizon of $>=$ 10 cm thickness, which contains at least 20 or 35% *SOM* (12 or 20% *SOC*) depending on the prevailing water regime and soil texture [17, 40]. While the German classification identifies organic sites by an organic layer $>=$ 30 cm, which begins within the first 70 cm below the soil surface and has an *SOM* content of 30 % ($SOC>=15~\%$)[2]. Therefore, the IPCC definition includes peatland sites according to the German definition plus a variety of other organic soils that are not included in the peatland German definition [7] (Table 2).

**Table 2:** Comparison of the peatland definition according to the IPCC (“IPCC definition”) and the organic soil definition according to the German soil classification system (“German *KA*5 definition”).

IPCC definition of peat soils	German *KA*5 definition of organic soils	*KA*5 subtype	description of subtype	IPCC & *KA*5 match
1. Organic horizon$>=10 cm$, if$<20 cm$with $SOC>=12 \%$when mixed to depth of 20 *cm* 2. Never water saturated for more than a few days,$SOC>20 \%$($SOM>35 \%$) 3. Episodically water saturated and a. Clay proportion$=0 \%$:$SOC>=12 \%$($SOM>=20 \%$) b. clay$>=60 \%$: $SOC>=18 \%$($SOM>=30 \%$) c. Intermediate clay amount: intermediate, proportional amount SOC	organic horizont$>=30 cm$consiting of peat (H-horizon) containing$SOM>=30 \%$	Anmoorgley	1. Organic horizont$<40 cm$with$SOM>=15 -<30 \%$ 2. Subject to saturation by ground- or backwater close to terrain surface	no
		Moorgley	1. Peat horizont$>=10-<30 cm$with$SOM>=30 \%$($SOC>=15 \%$) 2. Long-term saturated by groundwater close to the terrains surface	yes
		Hochmoor	1. Natural peatland 2. Develop under influence of rainwater 3. Peat horizon$<30 cm$with$SOM>=30 \%$($SOC>=15 \%$)	yes
		Niedermoor	1. Natural peatland 2. Develop under influence of ground- and/or floodwater accumulated at/above terrain surface 3. Peat horizon$<30 cm$with$SOM>=30 \%$($SOC>=15 \%$)	yes
		Erdhochmoor, Erdniedermoor, Mulmniedermoor	1. Cultivated peatland 2. Peat horizon$SOC>=15 \%$($SOM>=30 \%$), possibly disturbed by mineral layers but total thickness$<30 cm$	yes

“*KA*5 subtype” contains the subtypes of organic soils in the *KA*5, “description of subtype” their characteristics, and “IPCC & *KA*5 match” gives information about the subtypes compliance with both IPCC and *KA*5 regulations

**Fig. 1:**
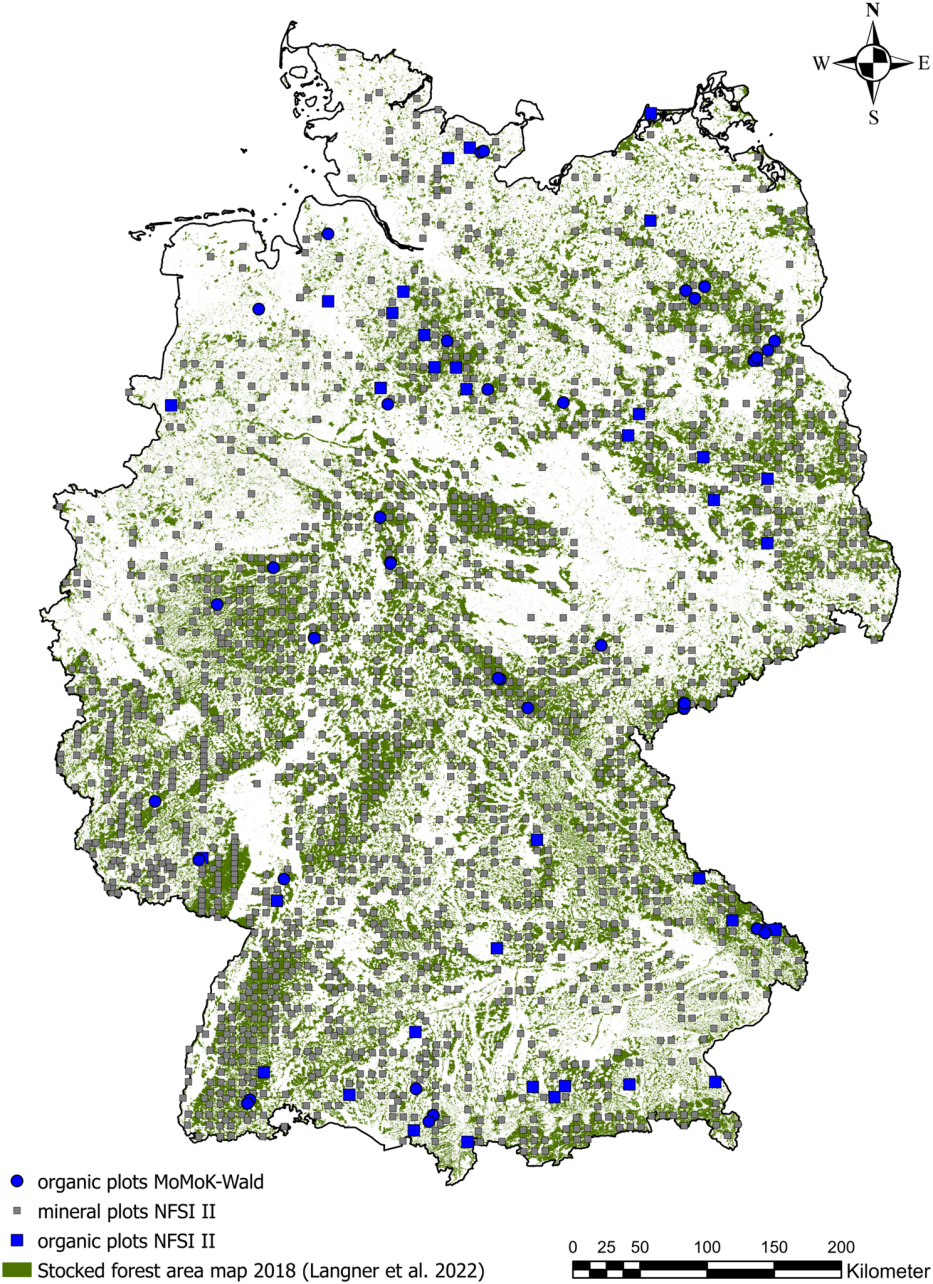
Overview of the sampling plots from NFSI II and $MoMoK-Wald$: Circles represent $MoMoK-Wald$ plots, while squares indicate NFSI II plots. Blue colour highlights peatland plots, grey colour refers to mineral soil plots. The green raster depicts a stocked forest area map of 2018 [52].

This selection resulted in a total of 65 forested peatland sites, consisting of 47 $MoMoK-Wald$ plots and 18 *NFSIII* plots. The remaining 1266 forested *NFSI* II plots are classified as mineral sites (Fig. 1).

All plots provided information on *DBH* (*cm*), tree age, geographical position, social class, canopy layer for all trees and *H* (*m*) for the five most representative individuals of each species, canopy layer and *DBH* class.

For trees whose diameter could not be measured at a height of 1.3 *m*, we used equation (*eq*.) 1 developed by Dahm [14] and adjusted by *NFSI* [14, 68] to convert the measured diameter into *DBH*. The results were divided by 10 to transform them to *DBH* in *cm*:

1 $$\begin{aligned} DBH = D_{\textrm{m}} + \frac{2 \times (H_{\mathrm{D_{\textrm{m}}}}-130)}{\tan _{\textrm{SP, C}}} \end{aligned}$$

where, *DBH* is the estimated diameter at breast height (*mm*), $D_{\textrm{m}}$ is the measured diameter (*mm*), $H_{\mathrm{D_{\textrm{m}}}}$ is the tree height in *m* at which $D_{\textrm{m}}$ was taken and $\tan _{\textrm{SP, C}}$ is the tangent specific to species and region.

To obtain the height for all trees in the plots - as the inventories only provide it for few representative trees - we fitted a non-lineal regression model for each species using the R-package *“forestmangr”* [9]. The model included the *H* and *DBH* delivered by the inventories:

2 $$\begin{aligned} H = \beta _\textrm{0} \times (1 - exp( - \beta _\textrm{1} \times DBH_\textrm{cm}))^{\beta _\textrm{2}} \end{aligned}$$

where *H* is the estimated height (*m*), $DBH_\textrm{cm}$ corresponds to the diameter at breast height (1.3*m*) in *cm* for all trees - with and without height information - and $\beta _\textrm{0}$, $\beta _\textrm{1}$ and $\beta _\textrm{1}$ are the coefficients fitted for each plot and species. We calculated the model coefficients applying the same formula for all trees with available field information on *DBH* and *H* for each species in the respective plot. The resulting plot- and species-specific coefficients were then applied to obtain the *H* of all trees. However, if the predictive capacity of these plot-specific models was not sufficient ($R^{2} < 0.7$), we fitted species-specific equations using *eq*. 2 and field data from all plots in the study area. If this model had sufficient predictability ($R^{2}> 0.7$) we calculated the *H* with these species-specific coefficients instead of the plot- and species-specific coefficients. Contrary, if it was still insufficient, we used the standard height function 3 by Sloboda et al. [80] and Riedel et al. [68] to estimate the *H* of the trees:

3 $$\begin{aligned} 0026; H = 1.3 + (H_\textrm{mean} - 1.3)*exp(k_\textrm{0}*(1 - \frac{DBH_\textrm{mean}}{DBH_\textrm{i}}))\nonumber \\0026; \quad *exp(k_\textrm{1}*(\frac{1}{DBH_\textrm{mean}} - \frac{1}{DBH_\textrm{i}})) \end{aligned}$$

where *H* is the estimated height (*dm*), $DBH_\textrm{mean}$ (*mm*) is the stands mean diameter at breast height (1.3 *m*), $DBH_\textrm{i}$ (*mm*) is the trees mean diameter at breast height (1.3 *m*), and $k_\textrm{0}$ and $k_\textrm{1}$ are species-group-specific coefficients. $H_\textrm{mean}$ refers to the stands mean height which is estimated by the following equation:

4 $$\begin{aligned}0026; H_\textrm{mean} \\0026;= \frac{(H_\textrm{g} - 1.3)}{exp(k_\textrm{0}*(1 - \frac{DBH_\textrm{mean}}{D_\textrm{g}})) *exp(k_\textrm{1}*(\frac{1}{DBH_\textrm{mean}} - \frac{1}{D_\textrm{g}}))} + 1.3; \end{aligned}$$

where $H_\textrm{mean}$ is the estimated mean height (dm), $DBH_\textrm{mean}$ (mm) is the stands mean diameter at breast height (1.3 m), $D_\textrm{g}$ (mm) is the diameter at breast height (1.3 m) of a tree representing the mean basal area of the stand, $H_\textrm{g}$ (dm) is the height corresponding to the *DBH* of a tree representing the mean basal area of the stand. The $k_\textrm{0}$ and $k_\textrm{1}$ are species-group-specific coefficients.

Height was recalculated for all trees that had a field height measurements, in order to homogenize the way all heights were extracted and reduce the variability caused by the method of height estimation.

To specifically describe tree height-diameter relationships of *A. glutinosa* and *B. pubescens* at mineral and peatland sites, we fit height curves as a function of *DBH* using the Peterson standard height function [50] and *H* and *DBH* of trees that had field measurements:

5 $$\begin{aligned} H = 1.3 + (DBH / (b_0 + b_1 * DBH_{cm}))^3 \end{aligned}$$

where *H* is the tree height in *m*, *DBH* is the diameter at breast height (1.3 *m*) in *cm* and $b_{0}$ and $b_{1}$ are the estimated coefficients. The functions were fit separately for each tree species and site type. The resulting parameter estimates were subsequently used for comparison between site types.

The *DBH* and *H* information was used to calculate the *WAG* by applying the 41, *DEU* equation through the R package *”TapeS”* [91], for all *B. pubescens* and *A. glutinosa* trees:

6 $$\begin{aligned} B = b_0 e ^{b_1}\frac{DBH}{DBH+k_1} e^{b_2}\frac{D_{03}}{D_{03}+k_2}H^{b_3} \end{aligned}$$

where *B* is the aboveground woody biomass in *kg*, *DBH* is the diameter at breast height (1.3 *m*) in *cm*, $D_{03}$ is the diameter at 30% of the trees height estimated by *TapeS*, *H* is the height in *m* and $b_{0}$, $b_{1}$, $b_{3}$, $k_{1}$ and $k_{2}$ are the coefficients of the function.

To compare 41, *DEU* with peatland- and species-specific equations, as well as species-specific equations, we applied the functions extracted from literature to the same dataset.

From this biomass estimate, we calculated the *C* stock by multiplying the *WAG* by a mean carbon content of 0.5 [39]. Carbon stock was extrapolated to hectares (*ha*) by summing the *C* mass of all trees of a species within each plot and dividing this total by the plot area (in *ha*).

### Statistical analyses

We compared attributes of *B. pubescens* and *A. glutinosa* trees in mineral and peatland forests, to determine if there are significant differences in tree growth at both sites. We used *DBH*, *H*, the coefficients of the height curves and the estimated *WAG* as independent variables to perform the comparison. As the data was not normally distributed, we tested for statistical significant differences of the mean using a Wilcoxon rank sum test for non-parametric data. In addition to comparing the overall mean of each independent variable, we segmented our data by *DBH* classes and tested the differences in *H* between mineral and peatland soils for each class. Normality was tested by performing a Shapiro-Wilk test.

Moreover, we analysed whether the 41, *DEU* allometric equation predicted similarly to the peatland or species-specific functions, selected through the literature review. We compared the biomass and carbon stock results of all equations using an ANOVA test to test if there are significant differences among the results. Following, we used a post-hoc Tukey-HSD test via the *”cars”* R package [22] to determine between which of the equations the significant differences occur. Specifically, we focussed on the results of 41, *DEU* compared to all other equations. In addition, we tested for significant differences in the *WAG* and *C* stock results depending on the site type for which their equation was developed using an ANOVA and a Tukey-HSD test. Therefore, before conducting the comparison, we grouped the equations in three categories: (1) “peat” − equations were parametrised from forests at peatland sites, (2) “mineral” − equations were parametrised from forests at mineral sites, (3) “mixed” − forest data used to develop the equation originated from mineral and peatland sites. We checked whether assumptions for both statistical tests were met by performing a Shapiro test for normality and a Levene test for common variance.

## Results

### Tree attributes at peatland and mineral sites

**Fig. 2:**
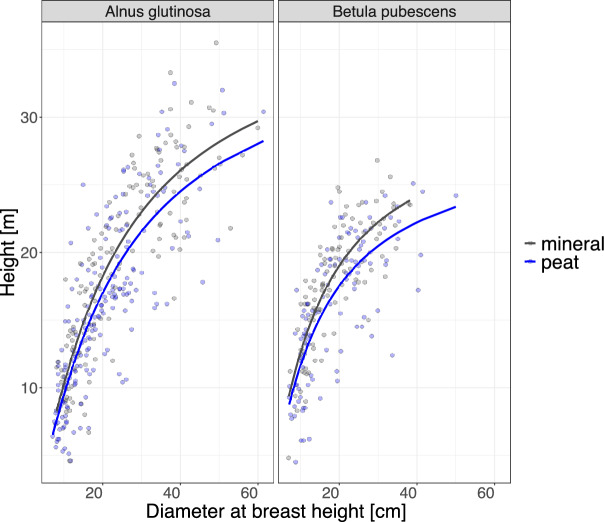
Relationship between the sampled tree height *H* (*m)* and diameter at breast height (1.3 *m*) *DBH* (*cm)* at peatland and mineral sites: The colours indicate the site type: Grey refers to mineral sites while blue refers to peatland sites. The lines of the height curves were fitted using Pettersons standard height equation $H = 1.3 + (DBH_{cm} / (b_0 + b_1 * DBH_{cm}))^3$ [50], for coefficients see Table 3.

We found that there are growth differences in *B.pubescense* and *A. glutinosa* forests located at peatlands and mineral sites (Fig. 2).

In peatland forests, both species exhibited significantly lower mean tree heights (*B. pubescens*: $$-$$1.87 m, *A. glutinosa*: $$-$$3.47 m) − at a comparable *DBH* − compared to mineral sites (Table 3). The trend was also shown in our fitted height curves, as the model’s $$b_0$$ and $$b_1$$ coefficients were significantly different for mineral and peatland sites (Table 3). Dissimilarity was stronger for *A. glutinosa*, where we found significant differences in mean *H* between site types for most *DBH* classes (Additional file 6). *H* diverged more strongly in higher *DBH* classes (for class $$>=$$ 30 cm we found a mean difference of 1.8 *m* *H*, while in class $$<$$ 30 cm it was of 1.03 *m* *H*). Contrary, *B. pubescens* only revealed significant results for *DBH* class 20 cm, where mean *H* differed by $$-$$2 m. Lower growth at peatland sites is also represented in the significantly lower mean estimated *WAG* of all equations at peatland sites for both species (p-value $$< $$ 0.001) (Table 3).

When analysing differences in mean *DBH* between mineral and peat sites, we found that only *A. glutionsa* displayed significant differences, with significantly larger trees located at mineral sites (Table 3). *A. glutinosa’s* mean *DBH* was averagely 4.9 cm lower in peatlands than at mineral sites, whereas for *B. pubescens* the difference was only 0.3 cm and not statistically different (Table 3).

**Table 3:** Results of the statistical comparison of tree attributes of *Alnus glutinosa* and *Betula pubescens* between mineral and peatland sites.

Species	Variable	Variable value at peatland sites	Variable value at mineral sites	p-value
*Alnus glutinosa*	mean *H* *m*	15.96	19.43	< 0.001
	mean DBH cm	20.50	25.44	< 0.001
	$b_0$	1.950999	1.849667	< 0.001
	$b_1$	0.3018018	0.2968738	< 0.001
	mean *WAG *$kg~tree^{-1}$	200.74	328.50	< 0.001
*Betula pubescens*	mean *Hm*	15.95	17.81	< 0.01
	mean *DBHcm*	19.02	19.33	0.3822
	$b_0$	1.284570	1.245175	< 0.001
	$b_1$	0.3307903	0.3213350	< 0.001
	mean *WAG *$kg~tree^{-1}$	175.65	168.26	< 0.001

“Species” displays the respective tree species, “variable” is the independent variable used for the comparison including mean diameter at breast height (*DBH* *cm*), mean measured height (*H* *m*), height curve coefficients (b_0_,b_1_) and estimated woody aboveground biomass (*WAG kg*).

### Comparison of biomass between equations

We found significant differences between the individual tree *WAG* for both *A. glutinosa* and *B. pubescens*, when comparing the results predicted by 41, *DEU* with the respective results of the peatland- or species-specific biomass equations sourced from literature (Additional file 2).

These differences to the results of 41, *DEU* occurred mainly between 41, *DEU* and equations developed for mineral sites (Additional file 2). However, in the case of *B. pubescens*, 41, *DEU* was also significantly different from a peatland-specific and a mixed-site equation (eq. 11, 2, *FIN*, eq. 26, *FIN*) (Additional file 2).

**Fig. 3:**
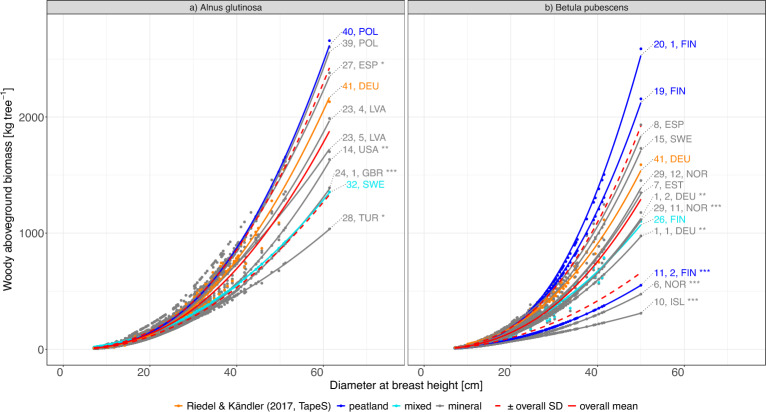
Estimated individual aboveground, woody biomass (*WAG*, $kg~tree^{-1}$) over diameter at breast height (1.3 *m*) *DBH* in *cm* for *Betula pubescens and Alnus glutinosa* at peatland sites: Orange means that the *WAG* was calculated through *eq* 41, *DEU* (*TapeS*, Riedel and Kändler [67]), the red solid line depicts the mean of all equations, the red dashed lines depict the upper and lower standard deviation around the mean, dark blue indicates an equation parametrised for peatlands, light blue means the equation was parametrised for mixed sites (mineral and peatland). Grey refers to equations that were exclusively calibrated for mineral sites. Asterisks highlight equations that were significantly different to 41, *DEU* (*** $p-value~<~0.001$, ** $p-value < 0.01$, * $p-value~<~0.05$).

When compared to the mean of the results of all equations, 41, *DEU* did not show significant differences for *A. glutinosa* (*p*-value $>~$1), exposing a similar general trend among the equations. For *B. pubescens* the overall mean of all equations and the predictions by 41, *DEU* differed significantly (p-value $<~$0.05). However, a DBH-class-wise analysis revealed the differences to be concentrated in lower *DBH* classes ($<~$20) (Additional file 7). Still, we observed that the predictions of 41, *DEU* slightly overestimated the mean individual tree *WAG* for both species. For *A. glutinosa* we found that the mean *WAG* was overestimated by 41, *DEU* by 7.27 $kg~tree^{-1}$, with increasing differences at higher diameters (Fig. 3a)). When the *DBH* surpassed 30 cm, the equation’s overestimation increased from −1.42$~kg~tree^{-1}$ for smaller trees to 51.5 $kg~tree^{-1}$ for larger trees ($\Delta$ mean overestimation: 50.08$~kg~tree^{-1}$) (Fig. 3a)). Contrary, for *B. pubescens* the 41, *DEU* equation had a more constant, but overall higher overestimation of 30.68 $kg~tree^{-1}$, when compared to the general mean (117.74 $kg~tree^{-1}$), with a mean overestimation about four times higher for larger trees compared to smaller trees (mean $WAG~DBH~<30$: $124~kg~tree^{-1}$, mean $\Delta$ for $DBH < 30$: $26.8~kg~tree^{-1}$; mean $WAG~DBH~>=30$: $676~kg~tree^{-1}$, mean $\Delta$ for $DBH~>=30$: $113~kg~tree^{-1}$) (Fig. 3b)).

Equation 41, *DEU* results for *B. pubescens* showed lowest mean differences in *WAG* to *eq*. 15, *SWE*, while the mean predictions for *A. glutinosa* by 41, *DEU* were closest to *eq*. 23, 5, *LVA* ( Additional file 2). The absolute and mean highest *WAG* was predicted by equations from sites that suited the ecological niche of the species (*eq*. 40, *POL*, *eq*. 27, *ESP*), or stands that had been fertilised in the past (*eq*. 20, 1, *FIN*). Whereas the lowest *WAG* estimates originated exclusively from stands at mineral sites (1) located in non-temperate climate zones (*eq*. 28, *TUR*, 10, *ISL*), (2) coppiced in the past (*eq*. 24, 1, *GBR*) or where (3) the compartmentalisation of the equation systematically excluded woody parts of the crown from the *WAG* (*eq*. 6, *NOR*). The *WAG* estimation for *B. pubescens* shows a higher coefficient of variation (*CV*) even in lower diameter ranges (*CV* = 1.28, standard deviation $SD~\pm ~162.08~kg$), compared to *A. glutinosa* trees (*CV* = 1.05, $SD~\pm ~203.51~kg$) (Fig. 3).

Moreover, we tried to compare the results of 41, *DEU* to the peatland-specific equations, yet we found high variability in their predictions. There was no distinct pattern in the variability of *WAG* estimates between the peatland-specific equations, as most of them predicted above (*eq*. 40 *POL*, *eq*. 20, 1, *FIN*, *eq*. 19, *FIN*) and below (*eq*. 32 *SWE*, *eq*. 11, 2 *FIN*) the overall standard deviation (Fig. 3).

Despite the lack of a clear trend within the peat-specific predictions themselves, there were differences in the predictive pattern among all site types (peat, mineral, mixed). The results of the peatland-specific equations for *A. glutinosa* showed a significant dissimilarity to results predicted by mineral and mixed sites equations (peat vs. mineral-specific: *p*-value $<0.001$, peat vs. mixed: *p*-value $<0.001$)(Additional file 3), while for *B. pubescens* *WAG* predictions by peat-specific equations differed significantly from mineral-specific equations (peat vs. mineral: $p-value < 0.001$).

### Comparison of carbon stocks between equations

For both species the *C* stocks calculated by 41, *DEU* were not significantly dissimilar to the overall mean (*A. glutinosa*: *p*-value $>=1.0$, *B. pubescens*: *p*-value $>=0.9$), nor any of the other equations results (Additional file 4).

**Fig. 4:**
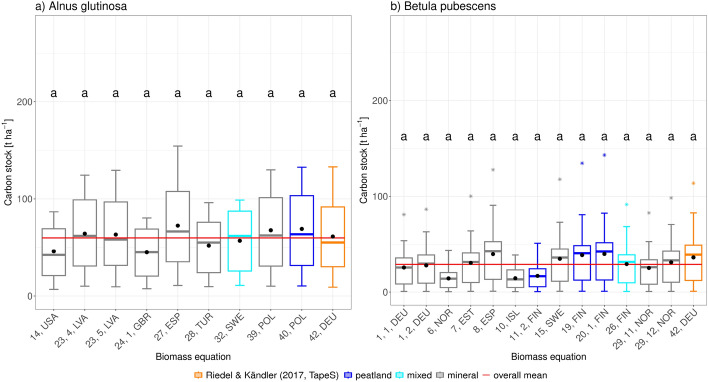
Carbon (*C*) stocks per hectare by biomass equation for and *Betula pubescens* and *Alnus glutinosa*: Orange means that *C* stock was calculated using *eq*. 41, *DEU* (*TapeS-*package, Riedel and Kändler [67]), the red solid line depicts the mean of all equations for peatland, black solid lines is the mean of all equations for mineral sites, dark blue indicates an equation parametrised for peatlands, light blue means the equation was parametrised for mixed sites, grey indicates equations that exclusively originate from mineral sites. Letters show significant differences between the groups.

With a mean of 36.16 $t~C~ha^{-1}$ the 41, *DEU* based *C* stock of *B. pubescens* stands was averagely 7.33 $t~C~ha^{-1}$ higher than the overall mean of 28.82 $t~C~ha^{-1}$ (Fig. 4b)). Meanwhile the mean *C* stock for *A. glutinosa* stands predicted by 41, *DEU* (61.35 $t~C~ha^{-1}$) exceeded the overall mean of 59.81 $t~C~ha^{-1}$ by 1.55 $t~C~ha^{-1}$ (Fig. 4a)). The overall mean *C* stocks estimated by 41, *DEU* were similar to those estimated by equations for forests located at both peatland and mineral sites (Fig. 4). Therefore, our results showed that 41, *DEU* was capable of correctly predicting the *C* stock for both species, in mineral and peatland sites.

The mean *C* stocks of *A. glutinosa* across all equations were higher than the mean *C* stocks for *B. pubescens* by 31.99 $t~C~ha^{-1}$, with *B. pubescens* estimations having a higher variability (*B. pubescens* *CV* = 0.31, *A. glutinosa* *CV* = 0.16). The *C* stocks of *B. pubescens* stands showed outliers for most of the biomass equations; while for *A. glutinosa* there were no outliers present in any of the equations (Fig. 4). It is important to note that for both species there were no outliers when calculating the *C* stocks using the 41, *DEU* equation (Fig. 4).

We did not observe any pattern in the predictions of the *C* stock between the peatland, mineral, or mixed equations, as there was no significant dissimilarity between the groups (*p*-value $>~0.05$) for both species (Additional file 5).

## Discussion

### Tree attributes at peatland and mineral sites

We found that the mean tree *H* and the height curve predictions for *A. glutinosa *and *B. pubescens* stands were lower for peatlands compared to mineral sites. This finding supports our first hypothesis that growth dynamics differ between peatland and mineral sites and is consistent with the significant difference we found in *WAG* predictions of peatland-specific equations compared to equations originating from mineral sites (Additional file 3). However, there were no significant differences in *DBH* between the two site types for *B. pubescens*. Yet, the differences in tree attributes resulted in significantly lower estimated mean *WAG* at peatland sites for both species. These results were partially consistent with findings of Repola et al. [66] and Hytönen [35] who both observed significant differences in height and *DBH* growth of *Betula pendula* and *Betula pubescens* for mineral and peatland sites in Finland. Anadon-Rosell et al. [4] and Rodríguez-González et al. [72] observed changes in the biomass accumulation of rewetted *Alnus spp.* stands, where they found that differences in the water regime of the site could affect tree growth. Therefore, we consider that the differences we observed in growth could be related to the biochemical processes resulting from changes in the water table [37, 41, 53, 66, 70]. Both, a high water table causing low oxygen availability, as well as a low water table that results in a lack of water, hinder photosynthesis, negatively affecting growth [53]. In addition, a high water table results in anoxic conditions that slow down decomposition rates, cause a lower availability of nutrients, and limit the penetration of the root system [10]. This leads to reduced growth rates compared to mineral sites, even if the species adapts to these conditions [70, 72].

Yet, the *WAG* results by peatland-specific equations were highly variable, making it difficult to identify predictive trends associated with the lower growth rates and smaller tree heights characteristic of peatlands. This discrepancy could arise from differences in the hydrological regimes of the datasets used to parametrise the equations [41, 53, 66, 70], or from the fact that most peatland equations rely solely on DBH to predict biomass and DBH for *B. pubescens* did not differ between site-types in our study (Table 3, Table 1). Therefore, we cannot conclusively negate or confirm that peatland-specific equations will estimate lower *C* stocks compared to our mineral-site specific 41, *DEU* equation, even though we found differences in tree attributes in our data.

### Comparing biomass and carbon stocks between equations

We found that the carbon stock predictions by 41, *DEU* for *Alnus glutinosa* and *Betula pubescens* were similar to the mean prediction of all other equations, making it reliable for estimating the *C* stock in German mineral and peatland soils on a large scale. This contradicts our initial hypothesis that 41, *DEU* would not be applicable to peatland forests of *A. glutinosa* and *B. pubescens* without adjustments. For *A. glutinosa* 41, *DEU* also delivered reliable estimations for the individual tree *WAG*, as the results were similar to the overall mean. Yet, the *WAG* predictions of *B. pubescens* differed significantly from the overall mean of all equations. However, this was not reflected in the *C* stocks as the dissimilarities originated from deviations in the *WAG* of smaller diameter classes ($DBH~<~20$), while most of the species’ basal area per hectare (and thus the biomass) is concentrated in higher diameter classes (Addition file 7). Thereby, the suitability of 41, *DEU* for predicting *C* stocks on stand level remained unaffected, since possible misestimations for smaller trees do not significantly effect the total estimates per hectare (Additional file 7). Nevertheless, the absence of significant differences in the larger *DBH* classes ($DBH~>~40$), despite the greater deviation among results, could be due to their small number of observations and their high variability, which may limit the data’s ability to reveal true differences. Therefore, we recommend restricting the use of 41, *DEU* for *B. pubscens* to large-scale *C* assessments as its predictability to calculate *WAG* for trees and stands in small diameter classes is limited.

The comparatively good predictability of 41, *DEU* could be related to the lack of differences in growing conditions between mineral and peatland site conditions. In the case of *B. pubescens*, these similarities could be enhanced, as *DBH* and *H* across most *DBH* classes - with the exception of class 20 cm - were not significantly different. This could be related to the fact that the sites in our study area most likely include a large proportion of peatlands in transition to mineral sites, as peatlands are often drained to enable forestry [7, 83]. Even though, the stands in our study are situated in peatlands, they likely represent a mixture of true peat forests and stands growing under conditions more characteristic of mineral soils. In pristine peatland ecosystems, the water table functions as the main factor limiting tree growth [4, 41, 66]. However, drainage can promote tree growth, as the soil transitions towards aerobe conditions more similar to those of mineral soils, which increase nutrient availability and root growth [5, 48, 53, 66]. As the majority of our peatlands sites are not pristine, we can consider that their growth conditions could align more with the original data 41, *DEU* was based on. This could explain why it currently functions effectively in peatland environments. However, the suitability of the equation for predicting *WAG* and *C* stocks may change in the future as peatland management increasingly prioritises the provision of ecosystem services such as carbon sequestration, biodiversity, and resilience[7, 47]. The resulting efforts to restore peatlands and the ongoing negative effects of climate change could alter current peatland forests dynamics [7, 64], thereby deviating from those of mineral sites for which TapeS was calibrated. Consequently, the accuracy of *WAG* estimations made by 41, *DEU* may decline over time. To ensure continued reliability, there is a growing need for more spatial and temporal data to validate and potentially recalibrate the model to include a differentiation between natural peatlands and altered peatland ecosystems.

The *WAG* and *C* predictions varied considerably between the equations, reflecting differences in the underlying datasets [73, 95]. This is represented in the upper and lower *WAG* and *C* estimates, which correspond to the particular conditions for which the equations were parameterised. For example, the highest *WAG* estimates for *A. glutinosa* came from peatland stands, where it inhabits its ecological niche [16, 61, 78], whereas the lowest *WAG* for *B. pubescens* was produced by *eq*. 10, *ISL* derived from extreme boreal growing condition [31]. The effects of stand treatment are evident in the results of *eq*. 20, *FIN* which returned the highest *B. pubescens* estimates, probably because the original stands growth was enhanced by drainage and fertilisation [33]. Moreover, differences in the definitions of the tree compartments could account for some of the variability observed in our data. For example, the exclusion of the “foliage” compartment leads to the underestimation of woody aboveground biomass calculated by 6, *NOR* since they compartmentalise in “stem” and “crown”, whereby the latter included woody and non-woody parts of the tree crown. Both species showed greater variability in *WAG* estimates with increasing DBH, which is likely because most of the equations were calibrated for smaller diameters than those represented in our study (Additional file 1). Hence, these equations represent the growth dynamics of young, site-adapted trees, which are characterised by higher growth rates [85]. Applying them beyond their original *DBH* ranges can reduce accuracy and cause systematic misestimations, thereby increasing the variability [12, 73, 95].

### Implications for peatland forest management and carbon sequestration

In terms of *C* management of forested peatlands, *A. glutinosa* stands show higher *C* stocks compared to *B. pubescens*, suggesting that there could be a higher potential for *C* sequestration. In addition, it is important to notice that the highest *WAG* estimations of peatland-specific equations for *A. glutinosa* originate from functions for rewetted or intact peatlands, while highest estimations for *B. pubescens* originate from drained peatland. This complies with the respective ecological niche that these species usually occupy in a peatland ecosystem and the difference between the two species in their reaction to different hydrological regimes[4, 61, 72]. *A. glutinosa* trees show a greater adaptability to wet site conditions and, particularly, changes in the water table [4, 16, 21, 25, 61, 72, 78]. This enables *A. glutinosa* to grow even when the root system of e.g. *B. pubescens* cannot adapt any more [16, 78]. However, the economic use of *A. glutinosa* wood has declined in recent centuries, restricting its occurrence mainly to heavily water-influenced riparian or peatland forests where it has a competitive advantage over other native species [38, 61]. These areas offer optimal growth conditions and remain largely unmanaged due to their inaccessibility and protected status, allowing undisturbed accumulation of substantial *C* stocks [7, 38, 61].

With regard to forest management, we can deduct that as peat restoration progresses, *Alnus glutinosa* may be a more site-adaptive species. For peatland areas undergoing transition toward restoration, or for sites that are not designated for active restoration and rewetting, promoting the establishment of A. glutinosa could be beneficial. This species is well adapted to fluctuations in the hydrological regime and may also provide positive effects on biodiversity [19, 21]. It is likely to adapt more easily to environmental changes and is therefore suitable for management objectives aimed at developing multifunctional peatland forests with a more stable carbon pool in the living peatland biomass. However, it is important to note that the establishment of forest stands cannot replace the ecological and climatic benefits of restoring and rewetting peatlands [48].

## Conclusions

Equation 41, *DEU* sufficiently estimates the biomass and carbon stock of peatland forests in Germany in their current state. However, with the growing efforts of peatland restoration but also with progressing climate change, site conditions in peatlands may change, potentially causing 41, *DEU* to loose accuracy in the future. Therefore, we recommend restricting the use of the 41, *DEU* equation to applications at broader, regional scales, as on a local scale in individual peatland forests with specific ecological conditions or at the individual tree level it is more appropriate to utilize site- and species-specific equations. Moreover, we were able to confirm in this study that tree growth dynamics at peatland and mineral sites differ in some aspects. Yet a more thorough analysis may be possible after repeated collection of stand data within the framework of NFSI III, which is currently taking place. Future research is needed to investigate the impact of the interrelation of climate change, changing peat conditions, and peatland restoration efforts on tree growth.

## Supplementary Information

Additional file 1: Biomass equations and coefficients: Additional file 1 displays all biomass equations and parameter values used for the comparison with 41, *DEU*. Biomass equations are reportet for all available biomass components (“comp”) where “AGB” refers to the total aboveground biomass, “SW” is stemwood ”SB” is stemwood bark, ”STW” is stumpwood ”STB” is stumpwood bark, ”FWB” is finewood NDL” is foliage. The “ln” is the natural logarithm and “log10” the 10-based logarithm. Columns “a”, “b”, “c”, “d”, “e”, “f”, “g”, “h”, “I”, “j”, “k”, “m”, “TSL”, “u_k” and “e_ki” provide parameter values. The IDs refer to the subsequent number of the paper used for the figures (“paper_ID”), the number of the equation in the respective paper (“func_ID”) and the ID used to refer to the equation in this study (“eq_ID”). The corresponding reference in this publication can be found in Table 1. The respective equation is provided in column “equation”. Column “peat” provides information about the site type the equation was parameterised for with “yes” meaning peatland, “no” meaning mineral sites and “partly” meaning the parameterisation included both, mineral and peatland sites. Additional information like units of the diameter (“DBH”), height (“H”) and biomass (“B”), number of sampled trees (“N”), coefficients of determination (“$\hbox {r}^2$/AIC”), and range of diameter (“DBH”), height (“H”) and age (“age”) of sampled trees are reported if available. Information on the respective reference are displayed in “author”, “tittle”, “year” and “DOI”.

Additional file 2: Results of the Tukey-HSD test for biomass by equation: Additional file 2 displays the results of the Tukey-HSD test comparing the *WAG* results of all biomass equations per species (“species”) for significant differences. Column “contrast” shows which equations results were tested against which, while “term” displays the variable the groups originate from, in this case the equations IDs (“eq_ID”). Further, the table contains the difference in means (“diff”), the confidence levels (“conf.low”, “conf.high”) and the adjusted p-values (“adj.p.value”) for all possible pairs.

Additional file 3: Results of the Tukey-HSD test for biomass by site type: Additional file 3 displays the results of the Tukey test comparing the *WAG* results of equations which were parametrised for peatland or mineral plots for significant differences. The species for which the differences were tested is listed in “species”. Column “contrast” shows which site type was tested against which, where “yes” means the equation was parametrised for peatland, “no” means the equation was parametrised for forests on mineral soils and “partly”, refers to equations that were developed with data from mineral and peatland sites. The column “term” displays the variable the groups originate from, in this case the equations peat status (“peat”). Further, the table contains the difference in means (“diff”), the confidence levels (“conf.low”, “conf.high”), and the adjusted p-values (“adj.p.value”) for all possible pairs.

Additional file 4: Results of the Tukey-HSD test for carbon stocks by equation: Additional file 2 displays the results of the Tukey-HSD test comparing the *C* stock results of all biomass equations per species (“species) for significant differences. Column “contrast” shows which equations results were tested against which, while “term” displays the variable the groups originate from, in this case the equations IDs (“eq_ID”). Further, the table contains the difference in means (“diff”), the confidence levels (“conf.low”, “conf.high”) and the adjusted p-values (“adj.p.value”) for all possible pairs. 

Additional file 5: Results of the Tukey-HSD test for carbon stocks by site type: Additional file 5 displays the results of the Tukey-HSD test comparing the *C* stock results of equations which were parametrised for peatland or mineral plots for significant differences. The species for which the differences were tested is listed in “species”. Column “contrast” shows which site type was tested against which, where “yes” means the equation was parametrised for peatland, “no” means the equation was parametrised for forests on mineral soils and “partly” refers to equations that were developed with data from mineral and peatland sites. The column “term” displays the variable the groups originate from, in this case the equations peat status (“peat”). Further, the table contains the difference in means (“diff”), the confidence levels (“conf.low”, “conf.high”) and the adjusted p-values (“adj.p.value”) for all possible pairs.

Additional file 6: Results of the Welch Two Sample t-test and Wilcoxon rank sum test comparing the tree height by diameter class: Additional file 6 displays the results of statistical tests comparing the measured tree heights (*H*) in *m* grouped by diameter class (“DBH_class”) for *Alnus glutinosa* and *Betula pubescens* (“species”), *DBH* classes were assigned in steps of 10 *cm* (e.g. class 10: 0-10 *cm*, class 20: 10-20 *cm*). Further, the table contains the difference in means (“diff”), the adjusted p-values (“adj.p.value”) and the test used for the comparison, depending on the distribution of the data in the DBH class (“method”).

Additional file 7: Results of the Tukey-HSD test for individual tree woody aboveground biomass (*WAG*) by diameter class: Additional file 6 displays the results of the Tukey-HSD test comparing the woody aboveground biomass (*WAG*) in $kg~tree~^{-1}$ calcualted by 41, *DEU* to the overall mean *WAG* grouped by diameter class (“DBH_class”) for *Betula pubescens* (“species”). *DBH* classes were assigned in steps of 10 *cm* (e.g. class 10: 0-10 *cm*, class 20: 10-20 *cm*). “$n\_ha$” is the number of trees per hectare, “$BA\_m2\_ha$” is the basal area in $m^{2}$ per hectare, “$B\_t\_ha\_41\_DEU$” displays the mean biomass in tons per hectare calculated by 41, *DEU* and “$B\_t\_ha\_all\_eq$” the overall mean biomass per hectare across all equations. Further, the table contains the difference in means (“diff”), the confidence levels (“conf.low”, “conf.high”) and the adjusted p-values (“adj.p.value”) for all possible pairs.

## Figures and Tables

**Fig. 5:**
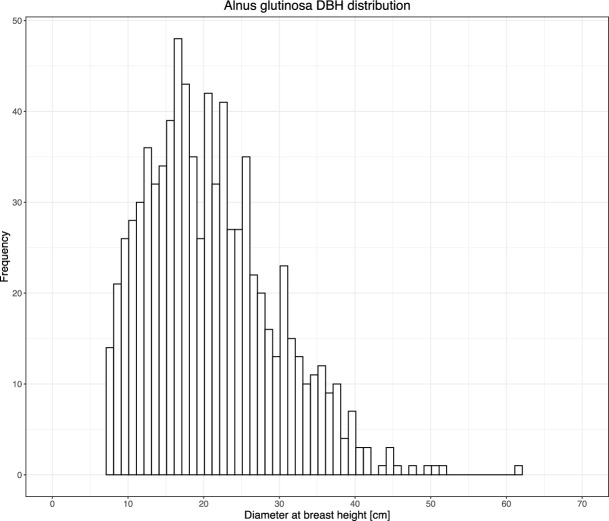
Histogram with the distribution of *Alnus glutinosa* by diameter at breast height in cm at peatland sites in Germany.

**Fig. 6:**
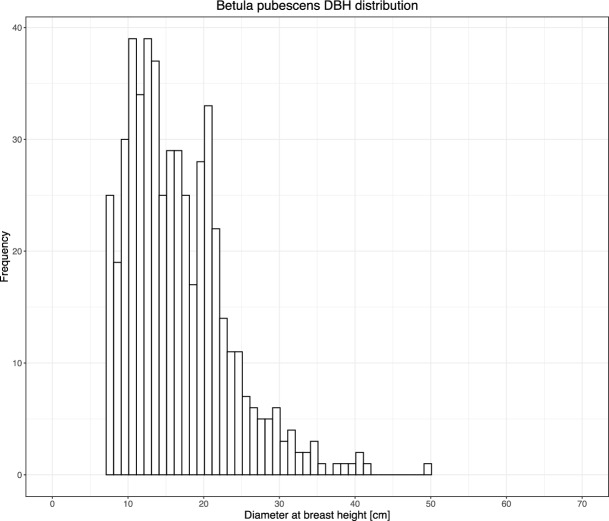
Histogram with the distribution of *Betula pubescens* by diameter at breast height in cm at peatland sites in Germany.

## Data Availability

No datasets were generated or analysed during the current study.

## Appendix A

See Figs. 5 and 6.
